# 

**Published:** 1882-02

**Authors:** 


### CONTENTS.

### *MISCELLANEOUS.*

PAGE.

Why We Fail.—Editorial, . . . . . . . . . . . . . 57

Misrepresentation. Ad. Lippe, M. D., Philadelphia, . . . . . 62

Specific Prescribing as against Pathological Prescribing. P. P. Wells, M. D., Brooklyn, N. Y., . . . . . . . . . . . . 66

Scientific Practice. [Extract.] . . . . . . . . . . 72

How to Use High Potencies. Stapf. . . . . . . . . . 73

Recent Resolution by the Royal College of Physicians, . . . . 73

Fatal Errors. Ad. Lippe, M. D., Philadelphia, . . . . . . . 75

Proving of Syphilinum. E. W. Berridge, M. D., London, . . . . 77

Homœopathy in Public Hospitals, . . . . . . . . . . 78

Physical Education Fallacies. [Extract.] . . . . . . . . 78

Notes from our Scrap-Book. E. J. L. . . . . . . . . . 82

*CLINICAL BUREAU:*

Intermittent Fever. C. F. Millspaugh, M. D., Binghamton, N. Y., . 88
Clinical Cases. E. W. Berridge, M. D , London, . . . . . . 90

Periscope, . . . . . . . . . . . . . . . . 98

*BOOK REVIEWS, NOTICES, ETC.*

Norton. Ophthalmic Therapeutics. Bœricke and Tafel, . . . . 102

Vilas. On the Ophthalmoscope. Duncan Bros , . . . . . . 103

Cate (W. M.), The Child of Promise, etc. H. B. Burnham & Co., . 104

Clapp (Otis & Son's.) Visiting List. Otis Clapp & Sons, . . . 104

Current Literature, . . . . . . . . . . . . . . 104

